# Supplementary material for: Virus-like Particles Carrying a Porcine Circovirus Type 2b Peptide Induce an Antibody Response and Reduce Viral Load in Immunized Pigs
Source: Vaccines (Basel). 2025 Dec 24;14(1):24. doi: 10.3390/vaccines14010024 (PMC12846673; doi:10.3390/vaccines14010024)

Supplementary Figure S1. Ct values of PCV2 detected by qPCR in nasal swabs and organs (Tonsils and lungs). Ct values obtained from nasal swab samples collected at 7, 14, and 21 days post-challenge (d.p.c.) in VLP-immunized pigs and non-immunized/challenged (n-i/c) animals (A). Ct values obtained from tonsil and lung samples collected at 21 d.p.c. in the same groups (B). Samples that tested negative by qPCR were uniformly assigned a Ct value of 40 for statistical purposes. Data were analyzed using one-way ANOVA, and statistically significant differences are indicated ($p < 0.05$; n/s: non-significant).

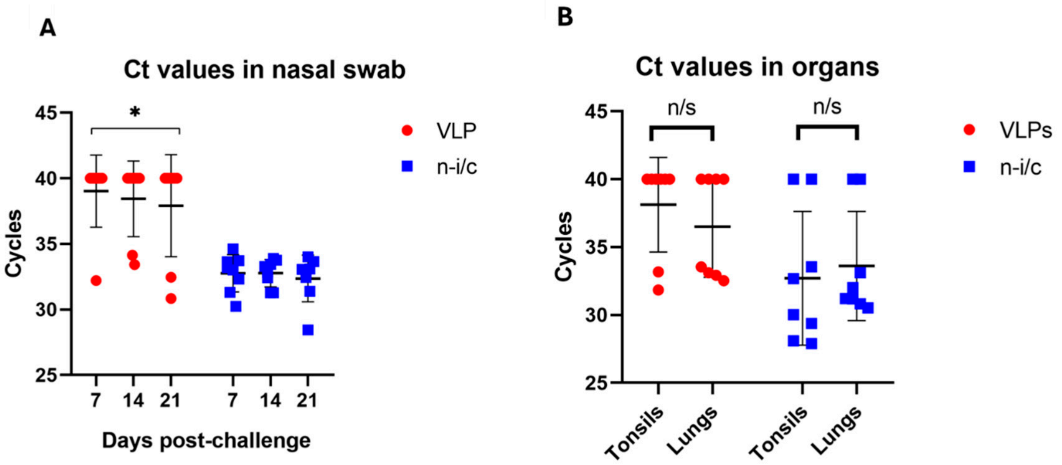

Supplementary Figure S2. Histopathological lesions found in PCV2-challenged individuals. Representative micrographs from an individual belonging to the non-immunized/challenged group are shown. (**A**) moderate lymphocytic interstitial pneumonia; (B) lung moderate necrosis and free heterochromatin; (**C**) moderate medial and intimal hypertrophy of the pulmonary artery; (**D**) mild lung interlobular edema; (**E**) moderate tonsil lymphoid hyperplasia; (**F**) moderate lymph node lymphoid hyperplasia.

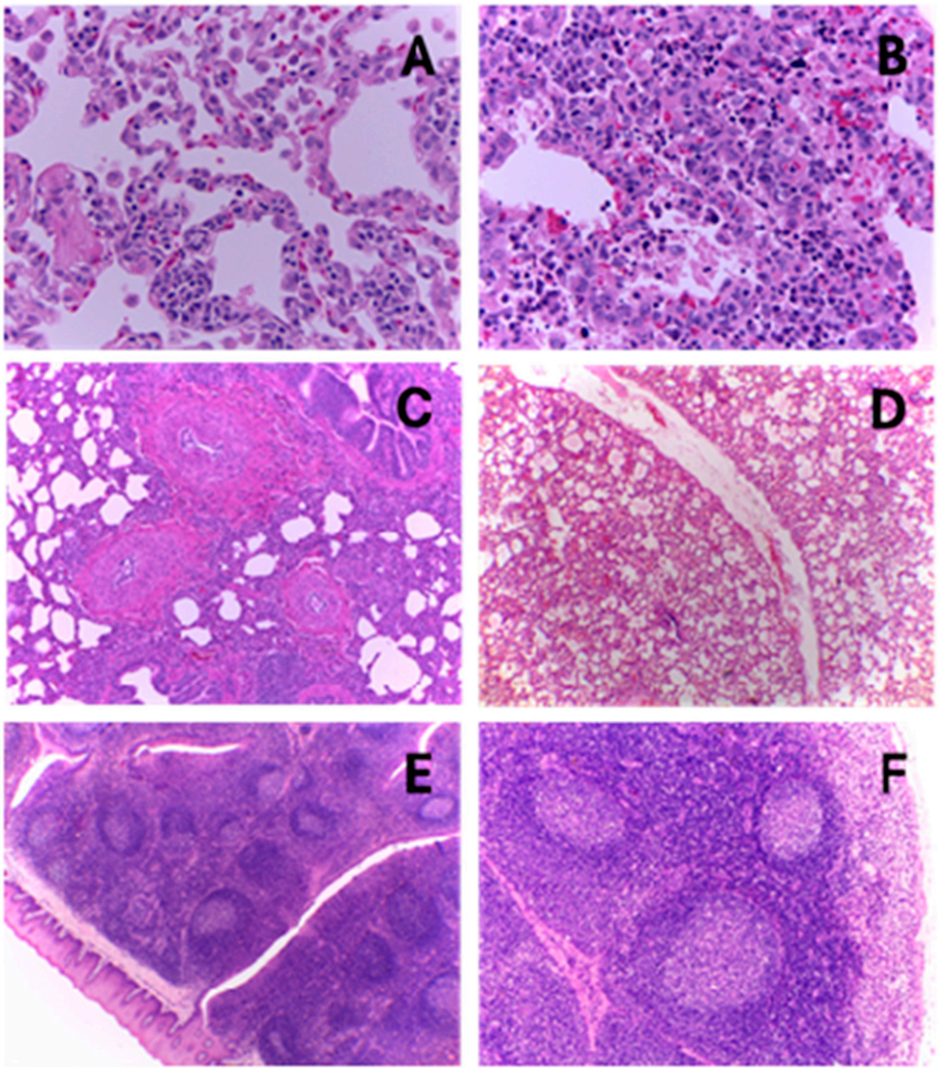

Supplement: Supplementary file 1 [file vaccines-14-00024-s001.zip › vaccines-3975089-supplementary.pdf]
